# In vitro evaluation of *Lactobacillus plantarum* as direct-fed microbials in high-producing dairy cows diets

**DOI:** 10.1093/tas/txz187

**Published:** 2019-12-21

**Authors:** Hugo F Monteiro, Ana Laura J Lelis, Virginia L N Brandao, Andressa Faccenda, Andre S Avila, Jose Arce-Cordero, Lorrayny G Silva, Xiaoxia Dai, Rasiel Restelatto, Perivaldo Carvalho, Leni R Lima, Antonio P Faciola

**Affiliations:** 1 Department of Animal Sciences, University of Florida, Gainesville, FL; 2 Departamento de Zootecnia, Universidade de São Paulo, Pirassununga, SP, Brazil; 3 Departamento de Zootecnia, Universidade Estadual de Maringa, Maringa, PR, Brazil; 4 Departamento de Zootecnia, Universidade Estadual do Oeste do Parana, Marechal Candido Rondon, PR, Brazil; 5 Centro APTA Bovinos de Corte, Instituto de Zootecnia, Sertaozinho, SP, Brazil; 6 Departamento de Zootecnia, Universidade Federal do Parana, Curitiba, PR, Brazil; 7 Departamento de Zootecnia, Universidade Federal do Mato Grosso, Cuiaba, MT, Brazil

**Keywords:** gas production, *Lactobacillus acidophilus*, *Lactobacillus plantarum*, * Saccharomyces cerevisiae*

## Abstract

The objectives of this study were: 1) to compare the effects of live yeast (LY), yeast fermentation product (YFP), a mix of *Lactobacillus acidophilus and Propionibacterium freudenreichii* (MLP), and *Lactobacillus plantarum* included as additives in dairy cows’ diets on in vitro ruminal fermentation and gas production (GP); and 2) to evaluate the effects of *L. plantarum* as direct-fed microbials (DFM) in dairy cows’ diets on in vitro ruminal fermentation, GP, nutrient digestibility, and N metabolism. Three experiments were carried out: Exp. 1 had the objective to compare all additives regarding ruminal fermentation parameters: an Ankom GP system was used in a completely randomized design, consisting of four 48 h incubations, and eight replications per treatment. There were eight treatments: a basal diet without additive (CTRL) or with one of the following additives: LY, YFP, MLP, or *L. plantarum* at four levels (% of diet Dry Matter (DM)): 0.05% (L1), 0.10% (L2), 0.15% (L3), and 0.20% (L4). In Exp. 2, a batch culture was used to evaluate ruminal fermentation, and CO_2_ and CH_4_ production using the same treatments and a similar experimental design, except for having 16 replications per treatment. Based on Exp. 1 and 2 results, Exp. 3 aimed at evaluating the effects of the *L. plantarum* on ruminal true nutrient digestibility and N utilization in order to evaluate the use of *L. plantarum* as DFM. The treatments CTRL, MLP, L1, and L2 were used in a replicated 4 × 4 Latin square design using a dual-flow continuous culture system. Data were analyzed using linear and nonlinear regression; treatment means were compared through contrasts, and L treatments in Exp. 1 and 2 were tested for linear, quadratic, and cubic effects. In Exp. 1, all treatments containing additives tended to reduce OM digestibility as well as reduced total volatile fatty acids (VFA) concentration and total GP. The YFP had greater OM digestibility than LY, and MLP treatment had greater total VFA concentration compared to *L. plantarum* treatments. In Exp. 2, additives reduced CO_2_ production, and there were no major differences in CH_4_. In Exp. 3, all additives reduced NH_3_-N concentration. In conclusion, pH and lactate concentration were not affected in all three experiments regardless of additive tested, suggesting that these additives may not improve ruminal fermentation by pH modulation; and *L. plantarum* may improve ruminal N metabolism when used as DFM in high-producing dairy cows’ diets, mainly by reducing NH_3_-N concentration.

## INTRODUCTION

Direct fed microbials (DFM) are live microbial additives that have been fed to high-producing dairy cows to modulate ruminal fermentation in order to enhance milk production (McAllister et al., 2011). Live yeast (LY) were one of the first DFM used for high-producing dairy cows because they are aerobic microorganisms that may improve ruminal anaerobiosis through oxygen scavenging and may metabolize ruminal lactate (McAllister et al., 2011). In a meta-analysis, Desnoyers et al. (2009) reported that LY increased milk production by increasing ruminal pH and total volatile fatty acids (VFA) while reducing lactate in the rumen. However, LY efficacy varies depending on dietary inclusion levels and diet composition, and LY may not survive long enough in the rumen (Beauchemin et al., 2006). As an alternative to LY, yeast fermentation product (YFP) containing yeast bioactive compounds and culture media, yet not viable yeast, has recently been used as additives for high-producing dairy cows because it has shown more consistency in its effects. Robinson and Erasmus (2009), summarized studies with YFP supplementation, and showed increases in milk production; however, some studies did not find positive responses when YFP was fed to high-producing dairy cows (Hristov et al., 2010; Leicester et al., 2016).

Another DFM fed to high-producing dairy cows are blends of lactic acid producing (LAB) and lactic acid utilizing (LAU) bacteria, which aim to increase ruminal pH, VFA production, and lactate utilization in the rumen (Philippeau et al., 2017). These blends, besides having LAU to metabolize lactate in the rumen, also contain LAB, which stimulates LAU proliferation through lactic acid production (Philippeau et al., 2017). Boyd et al. (2011) in an in vivo study supplementing a mix of *Lactobacillus acidophilus* and *Propionibacterium freudenreichii* (MLP) to high-producing dairy cows reported greater milk yield and apparent nutrient digestibility in animals receiving MLP, which according to the authors, may have been exacerbated due to heat stress. However, lack of responses are also observed when using these additives, and the reasons for these inconsistencies are not well understood (Raeth-Knight et al., 2007).

On the other hand, little is known about the effects of LAB supplemented alone as DFM in the diet of high-producing dairy cows. Although similar effects to MLP could be expected, this has not been thoroughly investigated. For feedlot cattle receiving high-grain diets, Beauchemin et al. (2003) reported that LAB (*Enterococcus faecium*), supplemented alone as DFM, had similar effects to ionophores on ruminal fermentation, increasing ruminal propionate concentration and pH, while decreasing ruminal protein degradation. Similar to these results, *Lactobacillus plantarum*, which is a common LAB silage inoculant, reduces silage protein degradation (Contreras-Goveaa et al., 2013; Muck et al., 2018), and therefore, may have the potential to increase the escape of ruminal undegraded protein in high-producing dairy cows.

Therefore, as objectives to this study, two preliminary experiments (Exp. 1 and 2) were carried out to compare the effects of LY, YFP, MLP, and *L. plantarum* included as additives in high-producing dairy cows’ diets on in vitro ruminal fermentation and gas production (GP); later, our main experiment (Exp. 3) was carried out to compare the effects of *L. plantarum* with MLP as DFM in high-producing dairy cows diets on in vitro ruminal fermentation, GP, nutrient digestibility, and N metabolism. We hypothesized that: 1) all additives would improve ruminal fermentation through an increase of ruminal pH; and 2) *L. plantarum* as DFM would increase VFA production and reduce ruminal protein digestibility.

## MATERIALS AND METHODS

### Location and Ethical Approval

Procedures related to the care and handling of the experimental animals were conducted under protocols approved by the University of Florida Institutional Animal Care and Use Committee.

### Preliminary Experiment 1

#### Experimental design and substrates

A basal diet was formulated to meet the dairy NRC recommendations (NRC, 2001), using as reference a Holstein cow producing 45 kg/d of milk, 90 DIM, and 680 kg BW (Table 1). Feed ingredients were ground to pass through a 2-mm screen in a Wiley Mill (model number 2; Arthur H. Thomas Co., Philadelphia, PA), and for chemical analysis the particle size was reduced to pass through a 1-mm screen using the same mill. Except the control treatment, which had no additive added to the basal diet, all other treatments had only a single additive added to their basal diet. Also, all additives were acquired in the solid form, and they were all individually added to the basal diet in a partial replacement to ground corn. Additives inclusion levels were determined following manufacturers recommendation for maximum responses, and *L. plantarum* inclusion levels were established in previous studies (A. P. Faciola and H. F. Monteiro, unpublished data). Therefore, the additive inclusion for each treatment was (% of diet DM): **CTRL** = control (no additive added); **LY** = 0.10% of live yeast; **YFP** = 0.10% of yeast fermentation product; **MLP** = 0.01% of mixed *L. acidophilus and P. freudenreichii*; **L1** = 0.05% of *L. plantarum*; **L2** = 0.10% of *L. plantarum*; **L3** = 0.15% of *L. plantarum*, **L4** = 0.20% of *L. plantarum*. Additive inclusion level, source, and composition are presented in Table 2.

**Table 1.** Ingredient and chemical composition of the basal diets used in the study (% of DM unless otherwise stated)^1^

Item	Basal diet, Exp. 1 and 2 (preliminary experiments)	Basal diet, Exp. 3 (main experiment)
Alfalfa hay	23.0	14.0
Corn silage	37.0	46.0
Ground corn	21.0	23.3
Solvent soybean meal 48% CP	16.5	14.2
Vitamin and mineral premix	2.50	2.50
*Chemical composition*		
OM	90.5	93.7
CP	15.9	16.0
RDP^2^	9.55	9.57
RUP^2^	6.35	6.40
NDF	28.6	31.7
Forage NDF	25.3	28.3
NFC^3^	43.6	43.5
Starch	27.2	29.3
Ether extract	2.37	2.50
NEL,^2^ Mcal/Kg of DM	1.55	1.55

^1^Ingredients were milled to pass through a 1-mm screen for chemical analysis and through a 2-mm screen for experiments.

^2^Estimated using the NRC (2001) model.

^3^NFC = 100 – (% NDF + % CP + % fat + % ash), according to the NRC (2001).

**Table 2.** Treatments and additive composition used in the preliminary Exp. 1 and 2

Treatment	Additive inclusion level (% of diet DM)	Composition
CTRL (control)	-	Basal diet only
LY	0.10%	*Saccharomyces cerevisiae* 7 1 × 10^7^ cfu/g
YFP	0.10%	Yeast fermentation product
MLP	0.01%	*Lactobacillus acidophilus NP*51 1 × 10^9^ cfu/g
		*Propionibacterium freudenreichii NP*24 2 × 10^9^ cfu/g
L1	0.05%	*Lactobacillus plantarum GB-LP1* 1.35 × 10^9^ cfu/g
L2	0.10%	*Lactobacillus plantarum GB-LP1* 1.35 × 10^9^ cfu/g
L3	0.15%	*Lactobacillus plantarum GB-LP1* 1.35 × 10^9^ cfu/g
L4	0.20%	*Lactobacillus plantarum GB-LP1* 1.35 × 10^9^ cfu/g

An in vitro GP experiment (preliminary Exp. 1) was performed to determine the kinetic parameters of GP, ammonia-nitrogen (NH_3_-N), lactate and VFA concentration after 48 h of incubation. The system used was an Ankom GP system (Ankom Technology, Macedon, NY), which was equipped with wireless pressure sensors that recorded electronically the total pressure of each bottle throughout the incubation. The experiment had the following design: there were eight experimental treatments, in which each treatment had two replications per incubation. In total, four incubations were performed, totaling eight replications per treatment. Dietary ingredients and DFM were individually weighted in each bottle, in which the latter were weighted in a Sartorius M2P microbalance (Göttingen, Germany). Bottles containing only ruminal content with no feed were used in all incubations as blanks.

### Media Preparation, Incubation Procedure, and Sample Collection

The ruminal/buffer solution was prepared according to Menke and Steingass (1988) except for the addition of sodium sulfite and L-cysteine as reducing agents (Paula et al., 2017). The resazurin solution was used as a color indicator for the redox potential. The buffer mineral solution was kept in a water bath at 39 °C and purged continuously with N_2_ until it became colorless. Ruminal content was collected 2 h after feeding from two rumen-cannulated steers (average BW of 550 kg). The diet of the steers consisted of 60:40 (forage:concentrate ratio), and was composed of orchardgrass hay, soybean meal (9.0% of total DM), and ground corn (31% of total DM). Mineral and salt blocks were provided ad libitum to the animals. The ruminal content was mixed with the buffer solution (1:2 v/v) in water bath at 39 °C under anaerobic conditions by flushing N_2_ (Menke and Steingass, 1988).

Each Ankom bottle (625 mL) contained 0.75 g (DM basis) of basal diet with or without the addition of an additive depending on the treatment. Bottles in this experiment were inoculated with 75 mL of buffered ruminal solution, also the bottle headspace was continuously flushed with N_2_. After inoculation, bottles were closed with the Ankom caps and placed in an air-ventilated shaker incubator (Innova 4400 incubator shaker; New Brunswick Scientific, Edison, NJ) under controlled temperature and agitation (39 °C and 80 RPM). The software for the Ankom system (Gas Pressure Monitor, Ankom Technology, New York) was set to record cumulative pressure every 5 min for 48 h. Valves of the Ankom caps were set to automatically release the gas when the pressure reached 3.4 kPa (Tagliapietra et al., 2011). The pH of the buffered ruminal solution was measured right before the incubation and it was measured again in each bottle at the end of the incubations with an Accumet portable AP61 pH meter (Fisher Scientific, Atlanta, GA). At the end of the incubation, subsamples of 8 mL of the final incubation media were collected and 2.0 mL of a 25% metaphosphoric acid solution was added for the determination of NH_3_-N, lactate, and VFA concentration, which are described below.

### Chemical Analysis

Feed ingredients were analyzed for DM (method 934.01; AOAC, 1990), ash (method 924.05; AOAC, 2012), CP (method 984.13; AOAC 1990), and dietary starch (enzymatic-colorimetric method; Hall, 2015). The Organic Matter (OM) was calculated as the difference between DM and ash contents. Feed ingredients were also analyzed for Neutral Detergent Fiber (NDF) according to Mertens (2002) and adapted for the Ankom^200^ Fiber Analyzer (Ankom Technology, Macedon, NY), and for Ether Extract (**EE**) according to AOAC (1990; method 920.85). Nonfiber carbohydrates (NFC) concentration of the feed ingredients were calculated using the NRC (2001) equation: NFC = 100 – (% NDF + % CP + % EE + % ash).

The concentration of NH_3_-N was determined similarly to Broderick and Kang (1980) with the exception that the analysis was performed in 96-well plates. Concentration of VFA was analyzed in a high-performance liquid chromatograph (HPLC; Hitachi L2400, Tokyo, Japan) according to Muck and Dickerson (1988). The HPLC was equipped with a UV detector set at 210 nm and a column Aminex HPX-87H set to 45 °C, in which 0.015M mobile phase sulfuric acid was used at a flow rate of 0.7 mL/min. The lactate concentration was analyzed with a D-lactic acid/L-lactic acid R-Biopharm kit through the procedure of Niederholtmeyer et al. (2010).

For cumulative pressure (kPa), the gas pressure was converted to units of volume (mL) using the ideal gas law, in which GP (mL) = (Pc/Po) × Vo, being Pc the cumulated pressure change (kPa) in the bottle headspace, Po the atmospheric pressure read by the equipment at the beginning of the measurement, and Vo the bottle headspace volume (545 mL). The final GP volumes were corrected by subtracting the final GP of the blank bottles. Fermentation rates (mL/h) and gas pool size (mL) were then calculated through the kinetics of GP, being gas pool size the possible maximum gas production predicted for each treatment. The OM digestibility was calculated based on total GP and diet chemical composition according to Menke and Steingass (1988).

### Preliminary Experiment 2

A second in vitro GP experiment (preliminary Exp. 2) was performed with the same treatments of preliminary Exp. 1 (basal diet: Table 1; additive and composition: Table 2). Serum bottles (160 mL) were used in a batch culture to measure fermentation pH, in vitro true OM digestibility, CH_4_ and CO_2_ production. The experiment was performed as follows: there were eight experimental treatments (the same of preliminary Exp. 1), in which each treatment had four replications per incubation. In total, 4 incubations were performed, totaling 16 replications per treatment. Dietary ingredients and DFM were individually weighted in each bottle and the latter weighted in a Sartorius M2P microbalance (Göttingen, Germany) as in preliminary Exp. 1. Blank bottles were also used in all incubations as in preliminary Exp. 1.

Each serum bottle (160 mL) contained 0.2 g (DM basis) of basal diet with or without the addition of an additive depending on the treatment. The media preparation was performed according to preliminary Exp. 1. Bottles were inoculated with 20 mL of buffered ruminal solution and the bottle headspace was continuously flushed with N_2_. After inoculation, bottles were closed with rubber stoppers and placed in the same air-ventilated shaker incubator used in preliminary Exp. 1 (Innova 4400 incubator shaker; New Brunswick Scientific, Edison, NJ) under controlled temperature and agitation (39 °C and 80 RPM). Each incubation lasted for 48 h and all bottles remained closed until the end of the incubation. The pH was also measured at the beginning in the buffered ruminal solution and in each bottle at the end of each incubation with an Accumet portable AP61 pH meter (Fisher Scientific, Atlanta, GA).

### Sample Collection and Chemical Analysis

At the end of the incubation, total pressure of each bottle was measured with a Druck DPI 104-IS Pressure Gauge (GE Measurements; Billerica, MA) and a subsample of 10 mL of gas was collected for CH_4_ and CO_2_ analysis. The concentration of both gases were determined in a Gow Mac thermal conductivity series 580 gas chromatography (Gow Mac Instrument, Bridgewater, NJ) equipped with a Porapak Q column (Supelco, 60 °C, 30 mL/min of helium 99.99% carrier gas). The final GP volumes were corrected by subtracting the final GP of the blank bottles.

The remaining fermented media was used for the determination of in vitro true OM digestibility. The media was dried in a ventilated oven at 55 °C, then NDF was analyzed according to Mertens (2002) and adapted to the Ankom^200^ Fiber Analyzer (Ankom Technology). The in vitro true OM digestibility was calculated according to Goering and Van Soest (1970): in vitro true OM digestibility (%) = (iOM – rNDF)/(iOM), in which iOM was the incubated OM and rNDF the residual NDF after 48 h of incubation minus the NDF content in the blank bottles.

### Main Experiment

#### Diets and experiment design

Based on the results of the preliminary experiments, the treatments MLP, L1, and L2 were selected in order to evaluate the ruminal effects of *L. plantarum* as DFM in a high-producing dairy cows’ diet on true nutrient digestibility and N utilization in a dual-flow continuous culture system (Exp. 3). A similar basal diet was formulated to meet the same animal requirements of the preliminary experiments (Table 1). Treatments were (% of diet DM): **CTRL** = control (no additive added); **MLP** = 0.01% of mixed *L. acidophilus and P. freudenreichii*; **L1** = 0.05% of *L. plantarum*; **L2** = 0.10% of *L. plantarum*. Additive source and composition are shown in Table 2. Diets were randomly assigned to eight dual-flow continuous culture fermenters in a replicated 4 × 4 Latin square arrangement with four 11-d experimental periods, consisted of 7 d for diet adaptation and 4 d of sample collections.

### Dual-Flow Continuous Culture System

Eight dual-flow continuous culture fermenters of 1,820 mL originally developed by Hoover et al. (1976) and recently modified by Benedeti et al. (2015), Silva et al. (2016), and Paula et al. (2017), were used in the study. Ruminal content was collected 2 h after feeding from two rumen-cannulated dairy cows (average 40 kg milk/d, and BW of 680 kg). The donor cows were fed a similar diet to that used for the preliminary experiments. Ruminal digesta was manually collected and strained through two layers of cheesecloth and approximately 15 liters of ruminal content were poured into prewarmed insulated vessels. The ruminal content from different cows were mixed in equal proportions at 39 °C and poured into the prewarmed fermenters until it reached the effluent limit. Ruminal content was continuously stirred by a central propeller apparatus set to 100 rpm and fermenters’ temperature was set to 39 °C. Each fermenter was fed 107 g/d of DM equally divided in two meals at 8:00 and 18:00 h.

Except for the addition of 0.4 g/L of urea to simulate urea recycling to the rumen, artificial saliva was prepared according to Weller and Pilgrim (1974), and it was infused at 3.05 mL/min. Liquid and solid dilution rates were adjusted daily to 11 and 5.5%/h, respectively, by adjusting artificial saliva input and liquid and solid removal rates. The pH of each fermenter was measured daily just before each feeding time using a portable pH meter (Thermo Scientific Orion Star A121).

Liquid and solid effluents were collected separately into two 4.3-liter plastic containers. During the first 7 d (adaptation period), the effluent containers were weighed daily before the morning feeding and the contents were discarded. On day 5, effluent digesta (liquid and solid) were homogenized and samples (500 mL) were collected to determine the background ^15^N abundance. Then, 0.1173 g of 10.2% excess of (^15^NH_4_)_2_SO_4_ (Sigma-Aldrich Co., St. Louis, MO) was infused in each fermenter to label the NH_3_-N pool. Saliva was reformulated and 0.077 g/L of enriched (^15^NH_4_)_2_SO_4_ (Sigma-Aldrich Co.) was added in replacement of isonitrogenous amounts of urea to maintain a steady-state concentration of ^15^N enrichment in the fermenters (Calsamiglia et al., 1996). Twenty-four hours before the first collection day and during the 4 d of sampling period, the temperature of the liquid and solid effluent containers were kept below 2 °C to prevent further microbial and enzymatic activities.

### Sample Collection

During the sampling days, pH of each fermenter was measured at 0, 1, 2, 4, 6, 8, and 10 h after the morning feeding using the portable pH meter previously described. On days 9, 10, and 11, samples (500 mL) of liquid and solid effluents from each fermenter were collected, homogenized, and stored at −20 °C for analysis of DM, ash, CP, NDF, and dietary starch. Additionally, a sample of 10 mL from the effluent containers were filtered through four layers of cheesecloth, part acidified at 0.1% with a 50% H_2_SO_4_ solution, and immediately stored at −20 °C for NH_3_-N, VFA, and lactate analyses. On days 10 and 11, a 10-mL ruminal content sample following the same acidification process was collected from the fermenter before the morning feeding, and at 1, 2, 4, 6, 8, and 10 h after the morning feeding from a composite of liquid and solid effluents for NH_3_-N and lactate concentration analysis. All samples were immediately stored at −20 °C for their respective analysis.

Later, acidified samples were centrifuged at 1,000 × g for 15 min at 4 °C, the supernatant was separated, isolated, and half was stored at −20 °C for ruminal NH_3_-N analysis. The remaining sample was centrifuged again at 7,000 × g for 15 min at 4 °C and filtered in cellulose acetate syringe filters (SF14485, Tisch Scientific) for VFA analysis. The nonacidified samples were centrifuged again at 7,000 × g for 15 min at 4 °C and the supernatant stored for lactate analysis following the same procedure used in the preliminary Exp. 1 by Niederholtmeyer et al. (2010). On day 11, the entire fermenter content was used for bacterial isolation as performed by Krizsan et al. (2010) and modified by Brandao et al. (2018).

### Chemical Analyses and Calculations

Feed, effluent, and bacterial samples were freeze-dried in a Labconco FreeZone 6 (Labconco Corporation, Kansas City, MO), and samples were analyzed for DM, ash, dietary starch, NDF, and EE according to Exp. 1, in which the OM content was calculated as the difference between DM and ash contents. Dietary starch was analyzed in the bacterial samples through the enzymatic-colorimetric method of Hall (2015) in order to account for the daily bacterial glycogen flow. The daily bacterial glycogen flow was quantified through marking the bacterial flow with (^15^NH_4_)_2_SO_4_ (Sigma-Aldrich Co.) and glucose analysis in the lyophilized bacterial samples.

The NFC content was calculated according to Exp. 1 based on the NRC (2001). Concentration of NH_3_-N, lactate, and VFA were also analyzed as in Exp. 1. Feed ingredients, bacteria, effluent digesta, and background samples were analyzed for total N and ^15^N enrichment with a CHNS analyzer (Dumas dry combustion method) connected to an isotope ratio mass spectrometer (Werner et al., 1999). Bacterial N and efficiency as well as N flows were calculated as described by Calsamiglia et al. (1996) and adapted from Reynal and Broderick (2005), respectively. The efficiency of N utilization (ENU) and bacterial efficiency were calculated as described by Brandao et al. (2018). The true nutrient digestibility calculations for DM, OM, CP, NDF, and dietary starch were performed according to Paula et al. (2017).

### Statistical Analyses

#### Preliminary experiments

Data were analyzed in a completely randomized design using the GLIMMIX procedure of SAS, with a model that included fixed effect of treatment and random effect of run. Means were compared through orthogonal contrasts, as follows: **Additive** = CTRL vs. other treatments; **MStype** = type of microbial source: yeasts vs. LABs (LY and YFP vs. MLP, L1, L2, L3, L4); **Yeast** = yeast type (LY vs. YFP); **LAB** = LAB source (MLP vs. L1, L2, L3, L4). The treatments L1, L2, L3, and L4 were also tested through contrasts for linear, quadratic, and cubic effects (**Llinear, Lquadratic, and Lcubic**). Only *P*-values of significant contrasts and tendencies are reported. For the kinetics of GP, an exponential model was used on SAS through the NLIN procedure. Least square means and SEM were reported, and significance was declared at *P* ≤ 0.05 and trends at 0.05 < *P* ≤ 0.10.

### Main Experiment

Data were analyzed using the GLIMMIX procedure of SAS as a replicated 4 × 4 Latin square design, with the model:

$$\begin{array}{l} Y_{ijkl}=Μ+\;L_{i}+\;P_{j}+\;F{{\left(L\right)}_{k}}_{i}+\;TR_{l}+\;E_{ijkl}, \end{array}$$

which Y_*ijkl*_ is the response variable, µ is overall mean, L_*i*_ is the effect of Latin square (i = 1 or 2), P_*j*_ is the effect of period (j = 1 to 4), F(L)_*ki*_ is the effect of fermenter (F) within square (k = 1 to 4), TR_*l*_ is the effect of treatment, and E_*ijkl*_ is the residual error. P and F(L) were considered random effects. Means were compared through orthogonal contrasts (CTRL vs. other treatments; MLP vs. L1, L2; L1 vs. L2). Least square means and SEM are reported for all the data with a significance declared at *P* ≤ 0.05 and trends at 0.05 < *P* ≤ 0.10.

Ruminal pH, NH_3_-N concentration, D-lactate, L-lactate, and total lactate concentrations were analyzed as repeated measures according to the model:

$$\begin{array}{ll} Y_{ijklm}=\; & Μ+L_{i}+\;P_{j}+\;F{\left(L\right)}_{ki}+\;TR_{l}+\;T_{m}\\ +\;TRT_{lm}+E_{ijklm}, \end{array}$$

which Y_*ijkl*_ is the response variable, µ is overall mean, L_*i*_ is the effect of Latin square (i = 1 or 2), P_*j*_ is the effect of period (j = 1 to 4), F(L)_*ki*_ is the effect of fermenter (F) within square (k = 1 to 4), TR_*l*_ is the effect of treatment, T_*m*_ is the effect of time (m = 1 to 16), TRT_*lm*_ is the interaction between treatment and time, and E_ijklm_ is the residual error. P and F(L) were also considered random effects. The covariate structures tested were: AR (1), ARH (1), CS, TOEP, TOEPH, UN, and VC. Based on the lowest AIC, the selected and used structures were: CS (pH) and AR(1) (NH_3_-N concentration, D-lactate, L-lactate, and total lactate). When there was no interaction between treatment and time, yet effects of treatment, data were compared using orthogonal contrasts (CTRL vs. other treatments; MLP vs. L1, L2; L1 vs. L2).

## RESULTS AND DISCUSSION

### Preliminary Experiment 1

There were no effects of additive inclusion (**additive contrast**: CTRL vs. other treatments) on final pH and gas pool size (Table 3). There was a trend for additive inclusion to reduce the fermentation rate (*P =* 0.10) and OM digestibility (*P =* 0.10), while total GP at 24 h (*P =* 0.04) and 48 h (*P =* 0.02) of incubation were reduced by additive inclusion compared to the CTRL treatment (Table 3). One possible reason for these negative effects in a short-term incubation may be due to a disruption in the ruminal microbial ecosystem, possibly causing reduction in cross-feeding, which is an important characteristic of the ruminal microbial ecosystem (Russell, 2002). Furthermore, studies with less than 30% starch levels in the diets have reported no increase in nutrient digestibility when yeast or lactobacilli were added to the diets since there were no abundant substrates for lactate production (Doreau and Jouany, 1998; Raeth-Knight et al., 2007; Hristov et al., 2010). Also, Williams and Newbold (1990) and Leicester et al. (2016) suggested that supplementation of microbial additives such as yeast may reduce total tract OM digestibility estimation because of an improvement in intestinal health, which could increase the endogenous secretion of OM to the intestine, leading to an underestimation of the true OM digestibility (Leicester et al., 2016).

**Table 3.** Effects of additives on OM digestibility and kinetics of gas production using an Ankom gas production system (preliminary Exp. 1)

	Treatments^1^										
Item	CTRL	LY	YFP	MLP	L1	L2	L3	L4	SEM	Contrasts of significance^2^	*P-*values of contrasts^3^
Final pH	6.07	6.08	6.08	6.08	6.10	6.11	6.08	6.10	0.11	Lcubic^†^	0.09
OM digestibility^4^, %	73.7	68.1	73.0	71.8	70.1	70.1	70.0	73.0	2.38	Additive^†^; Yeast	0.10; 0.03
Fermentation rate, h	0.06	0.06	0.05	0.05	0.05	0.06	0.06	0.05	0.01	Additive^†^; Lquadratic^†^	0.10; 0.08
Total GP_24 h_^5^, mL/g DM	220	192	205	204	200	205	202	213	9.05	Additive	0.04
Total GP_48 h_^5^, mL/g DM	256	224	236	245	236	236	227	247	11.0	Additive	0.02
Gas pool size, mL/g DM	325	292	318	324	305	306	300	328	21.0	Yeast^†^; Lcubic^†^	0.06; 0.09

^1^Additive in the basal diet for each treatment (% of diet DM): **CTRL** = control (no additive added); **LY** = 0.10% of live yeast (*S. Cerevisiae*); **YFP** = 0.10% yeast fermentation product (*S. Cerevisiae*); **MLP** = 0.01% of a mix of *L. acidophilus* and *P. freudenreichii*; **L1** = 0.05% of *L. plantarum*; **L2** = 0.10% of *L. plantarum*; **L3** = 0.15% of *L. plantarum*, **L4** = 0.20% of *L. plantarum*.

^2^Contrasts: **Additive** = CTRL vs. additives (CTRL vs. other treatments); **MStype** = yeast vs. LAB (LY and YFP vs. MPL, L1, L2, L3, L4); **Yeast** = yeast type (LY vs. YFP); **LAB** = LAB source (MLP vs. L1, L2, L3, and L4); **Llinear**, **Lquadratic**, and **Lcubic** = L1, L2, L3, and L4 were tested for linear, quadratic, and cubic effects.

^3^Significant differences were considered at *P* ≤ 0.05, and a tendency (†) was considered to be between *P >* 0.05 and ≤ 0.10.

^4^OM digestibility calculated according to Menke and Steingass (1988).

^5^Total gas volume produced after 24 and 48 h of incubation per gram of DM incubated.

In our study, which isolates the effects of the ruminal fermentation, we observed a tendency to reduce ruminal fermentation rate and OM digestibility in treatments containing the additives, while gas pool size was similar across treatments. This means that the amount of potentially fermentable substrates remaining after incubation was greater for the additive treatments, showing that the decrease in the OM digestibility may happen in the ruminal fermentation itself. The reduction in GP may have occurred because of the tendency to reduce OM digestibility, since GP from ruminal fermentation is positively correlated with OM digestion (Menke and Steingass, 1988).

Additive inclusion reduced total VFA concentration (*P =* 0.01), tended to increase acetate molar proportion (*P =* 0.09) and to reduce valerate molar proportion (*P =* 0.06; Table 4). The reduction in total VFA concentration for additive inclusion contrasts with studies using yeast (Desnoyers et al., 2009), although some studies with lactobacilli have also reported no effects on VFA concentration (Raeth-Knight et al., 2007; O’Brien et al., 2013). In the current study, the reduction in VFA is possibly due to the tendency for lower OM digestibility when additives were included, which limited the energy available for VFA production. The tendency for greater acetate and lower valerate molar proportions with the additive inclusion, may have happened due to the low lactate accumulation.

**Table 4.** Effect of additives on NH_3_-N and organic acids concentration using an Ankom gas production system (preliminary Exp. 1)

Item	Treatments^1^								SEM	Contrasts of significance^2^	*P-*value of contrasts^3^
	CTRL	LY	YFP	MLP	L1	L2	L3	L4			
NH_3_-N^4^, mg/dL	39.6	39.2	38.9	39.0	39.2	38.8	39.4	39.2	2.44	-	-
Total VFA, m*M*	120	108	105	118	99.8	103	91.7	92.5	6.17	Additive; LAB	0.01; <0.01
VFA, % of total VFA											
Acetate	48.9	50.6	51.1	50.6	51.5	47.7	50.4	49.8	2.52	Additive^†^; Lquadratic; Lcubic	0.09; 0.03; <0.01
Propionate	22.4	22.3	22.4	22.1	23.0	22.0	22.9	22.7	0.79	Lcubic	0.05
Butyrate	20.0	19.5	19.8	19.2	19.3	19.9	20.3	20.3	2.01	Llinear^†^	0.09
Valerate	4.58	3.99	3.22	3.83	3.01	4.26	3.19	3.81	0.60	Additive^†^; Lcubic^†^	0.06; 0.08
*Iso-*butyrate	1.30	1.24	1.13	1.51	1.16	1.15	1.09	0.99	0.16	LAB	0.02
*Iso-*valerate	2.76	2.29	2.34	2.79	2.06	2.30	2.09	2.39	0.39	LAB	0.05
Acetate:propionate	2.20	2.28	2.31	2.31	2.24	2.18	2.22	2.20	0.17	MStype; LAB	0.02; 0.01
BCVFA^5^, m*M*	3.93	3.83	3.63	4.38	3.16	3.56	2.91	3.14	0.40	LAB	<0.01
Lactate, m*M*	0.23	0.22	0.22	0.23	0.21	0.22	0.22	0.21	0.01	-	-

^1^Additive in the basal diet for each treatment (% of diet DM): **CTRL** = control (no additive added); **LY** = 0.10% of live yeast (*S. Cerevisiae*); **YFP** = 0.10% yeast fermentation product (*S. Cerevisiae*); **MLP** = 0.01% of a mix of *L. acidophilus* and *P. freudenreichii*; **L1** = 0.05% of *L. plantarum*; **L2** = 0.10% of *L. plantarum*; **L3** = 0.15% of *L. plantarum*, **L4** = 0.20% of *L. plantarum*.

^2^Contrasts: **Additive** = CTRL vs. additives (CTRL vs. other treatments); **MStype** = yeast vs. LAB (LY and YFP vs. MPL, L1, L2, L3, L4); **Yeast** = yeast type (LY vs. YFP); **LAB** = LAB source (MLP vs. L1, L2, L3, and L4); **Llinear**, **Lquadratic**, and **Lcubic** = L1, L2, L3, and L4 were tested for linear, quadratic, and cubic effects.

^3^Significant differences were considered at *P* ≤ 0.05, and a tendency (†) was considered to be between *P >* 0.05 and ≤ 0.10.

^4^NH_3_-N = ammonia nitrogen. ^5^BCVFA = Branched-Chain VFA (Isobutyrate + Isovalerate).

Contreras-Goveaa et al. (2013) reported that *L. plantarum* inoculums may improve silage and ruminal fermentation through a reduction in the silage of AA deamination and NH_3_-N concentration, and an increase on ruminal fermentation of microbial N, although ruminal VFA production is not altered. Leicester et al. (2016) reported that adding LY or YFP to high-producing dairy cows’ diet reduced total tract protein digestibility which may also be a consequence of reduced feed protein fermentation. In the preliminary Exp. 1, protein digestibility was not evaluated; however, additive inclusion did not change NH_3_-N concentration (Table 4). Lactate concentration was also not affected by additive inclusion (Table 4), possibly because different than in silage, the ruminal bacteria rapidly metabolize lactate if the diet used does not allow high lactate production (Weinberg et al., 2003), which may have happened in this experiment.

The type of microbial additive (**MStype contrast**: yeast vs. LAB treatments) affected acetate:propionate ratio (A:P), in which yeast treatments had in average greater ratio compared to LAB treatments (*P =* 0.02; Table 4). The difference in the A:P ratio may be due to differences between type of LAB treatments (**LAB contrast:** MLP vs. L1, L2, L3, L4 treatments; *P =* 0.01) in which MLP that is a mix of lactic acid producing and utilizing bacteria (*L. acidophilus* and *P. freudenreichii*) had greater A:P ratio than L1, L2, L3, and L4 treatments (*L. plantarum* treatments) and similar to yeast treatments (Table 4). Thus, these differences in A:P ratio for MStype correspond mainly to a LAB type effect than MStype itself. In general, these differences in A:P ratio were a result of the acetate molar proportion that tended to be greater for additive inclusion, and by a cubic response to L1, L2, L3, and L4 for both acetate and propionate molar proportions.

Yeast treatments (**yeasts contrast**: LY vs. YFP) differed in OM digestibility (*P =* 0.03), in which the YFP treatment had greater OM digestibility compared to the LY treatment. Yeast treatments also tended to have different gas pool sizes (*P =* 0.06) and YFP was greater than LY treatment as a consequence of the greater OM digestibility. Specifically in in vivo studies, YFP and LY have been shown to have similar effects on apparent total tract OM digestibility (Doreau and Jouany, 1998; Hristov et al., 2010; Leicester et al., 2016). However, these differences, together with the lack of responses in VFA concentration among yeast additives, may have happened because of an improved fermentation efficiency of LY treatment.

There was a LAB additive effect on total VFA concentration (*P <* 0.01), acetate:propionate ratio (*P =* 0.01), isobutyrate (*P =* 0.02), isovalerate (*P =* 0.05), and consequently on branched-chain VFA (BCVFA; *P <* 0.01), in which MLP treatment was always greater than L1, L2, L3, and L4 treatments (Table 4). Indeed, in in vivo studies using similar additives to MLP, treatments did not differ with the control diet in VFA concentration (Raeth-Knight et al., 2007; Philippeau et al., 2017) while in studies using pure *L. plantarum* the treatments either did not differ with a control diet or reduced total VFA concentration (Contreras-Goveaa et al., 2013; O’Brien et al., 2013). These results associated to the present study shows that the blend of MLP may be more advantageous in regards to total VFA compared to *L. plantarum* alone. O’Brien et al. (2013) also reported that *L. plantarum* produces H_2_O_2_ that inhibits methanogens activity and may promote H^+^ accumulation during fermentation, which would reduce the activity of H^+^ producing pathways (such as for acetate and butyrate). In fact, in our study additive inclusion tended to reduce total VFA concentration, and as the dose of *L. plantarum* increased the total VFA concentration reduced (L1 and L2 greater than L3 and L4). However, because MLP treatment had a mix of lactic acid producing and utilizing bacteria it did not affect the fermentation as seen in the pure *L. plantarum* treatments.

Acetate:propionate ratio was greater in *L. plantarum* treatments, which could be attributed to the numerically greater (*P* = 0.18) propionate molar proportion in *L. plantarum* treatments compared to MLP. On the other hand, BCVFA concentration is a result of both branched-chain AA oxidative deamination and decarboxylation during fermentation (Allison and Bryant, 1963) and cellulolytic bacteria uptake for branched-chain AA synthesis (Russell, 2002). As described earlier, *L. plantarum* has been reported to reduce protein degradation in the silage (Contreras-Goveaa et al., 2013), which could explain the lower BCVFA concentration for *L. plantarum* treatments while LAB additives did not differ in OM digestibility.

The polynomial responses for the *L. plantarum* treatments (L1, L2, L3, and L4) which had the same additive at different inclusion levels (0.05, 0.10, 0.15, and 0.20% of the diet DM) were mostly quadratic and cubic, with the exception for butyrate concentration that tended to have a positive linear response (*P =* 0.09). Ellis et al. (2016) also reported a tendency to a linear response for butyrate molar proportion; however, they used a lower *L. plantarum* inclusion level than in our study, which could have lowered their effects as well (e.g., butyrate molar proportion). *Lactobacillus plantarum* levels tended to cubically affect the final pH of fermentation (*P =* 0.09), gas pool size (*P =* 0.09), and valerate concentration (*P =* 0.08), and also tended to quadratically affect the fermentation rate (*P =* 0.08), which despite of being minimum changes these quadratic and cubic effects demonstrate that L1 and L2 may be a better DFM in high-producing dairy cow’s diet than L3 and L4.

### Preliminary Experiment 2

Additives reduced the CO_2_ production (mL/g DM) compared to the CTRL treatment (Table 5; *P =* 0.02) and tended to reduce CO_2_ production (mL/g dig. OM; *P =* 0.09). Similar changes were observed in the preliminary Exp. 1 when total GP at 24 h and 48 h of incubation and total VFA concentration reduced with additive inclusion. Therefore, we could confirm that the lower CO_2_ production may have been the reason for the reduction in total GP at 24 h and 48 h in the preliminary Exp. 1. The CO_2_ production is mainly driven by acetate and butyrate synthesis by ruminal bacteria (Russell, 2002), and although the acetate molar proportion tended to increase for additive inclusion in the preliminary Exp. 1, its total production was lower due to the lower total VFA concentration. No changes were observed for additive inclusion on CH_4_ production (mL/g DM or mL/g dig. OM).

**Table 5.** Effect of additives on ruminal fermentation parameters using batch culture as a gas production system (preliminary Exp. 2)

Item	Treatments^1^								SEM	Contrasts of significance^2^	*P-*value of contrasts^3^
	CTRL	LY	YFP	MLP	L1	L2	L3	L4			
Final pH	6.27	6.27	6.26	6.27	6.27	6.29	6.27	6.26	0.05	Lquadratic	0.04
True OM digestibility^4^, %	87.0	85.3	86.0	86.9	85.5	85.7	86.1	86.6	0.91	-	-
Gas production_48 h_^5^, mL/g DM											
CO_2_	63.1	60.7	60.9	61.3	61.2	60.7	61.8	58.3	1.97	Additive; Llinear^†^	0.02; 0.08
CH_4_	9.32	9.06	9.11	9.14	9.12	9.03	9.35	8.83	0.37	Lcubic^†^	0.09
Gas production_48 h_^6^, mL/g dig. OM											
CO_2_	80.3	78.7	78.4	78.2	79.1	78.0	79.6	73.6	2.29	Additive^†^; Llinear	0.09; 0.01
CH_4_	11.8	11.7	11.7	11.7	11.7	11.6	12.0	11.2	0.50	Lcubic^†^	0.07

^1^Additive in the basal diet for each treatment (% of diet DM): **CTRL** = control (no additive added); **LY** = 0.10% of live yeast (*S. Cerevisiae*); **YFP** = 0.10% yeast fermentation product (*S. Cerevisiae*); **MLP** = 0.01% of a mix of *L. acidophilus* and *P. freudenreichii*; **L1** = 0.05% of *L. plantarum*; **L2** = 0.10% of *L. plantarum*; **L3** = 0.15% of *L. plantarum*, **L4** = 0.20% of *L. plantarum*.

^2^Contrasts: **Additive** = CTRL vs. additives (CTRL vs. other treatments); **MStype** = yeast vs. LAB (LY and YFP vs. MPL, L1, L2, L3, L4); **Yeast** = yeast type (LY vs. YFP); **LAB** = LAB source (MLP vs. L1, L2, L3, and L4); **Llinear**, **Lquadratic**_,_ and **Lcubic** = L1, L2, L3, and L4 were tested for linear, quadratic, and cubic effects.

^3^Significant differences were considered at *P* ≤ 0.05, and a tendency (†) was considered to be between *P >* 0.05 and ≤ 0.10.

^4^Analyzed according to Goering and Van Soest (1970).

^5^Volume of total gas, CO_2_, and CH_4_ produced after 48 h of incubation for each gram of DM incubated.

^6^Total gas volume produced after 48 h of incubation per gram of digested OM.

*Lactobacillus plantarum* treatments (L1, L2, L3, and L4 treatments) quadratically affected the final pH of fermentation (*P =* 0.04), although differences were small. However, *L. plantarum* treatments tended to cubically affect CH_4_ production (mL/g DM and mL/g dig. OM; *P =* 0.09 and *P =* 0.07); had a linear response to CO_2_ production (mL/g dig. OM; *P =* 0.01), and tended to have a linear response to CO_2_ production (mL/g DM; *P =* 0.08). In studies evaluating microbial characteristics itself, *L. plantarum* has been reported to produce antimicrobial compounds, such as H_2_O_2_ and bacteriocins (Price and Lee, 1969; Zalán et al., 2005). In an in vitro study evaluating the time-dependent response for *L. plantarum* culture, O’Brien et al. (2013) reported that the longer *L. plantarum* is cultured the more H_2_O_2_ it produces and the more it reduces CH_4_ and CO_2_ production. In the same study, the *L. plantarum* culture that reduced CH_4_ and CO_2_ production the most, also reduced in vitro ruminal total VFA concentration, concluding that GP production and total VFA concentration were affected by either H_2_O_2_ or a bacteriocin. In contrast, Ellis et al. (2016), in a similar study evaluating in vitro ruminal fermentation (72 h) effects for *L. plantarum* at four different doses (0.5 *×* 10^5^, 1 *×* 10^6^, and 5 *×* 10^6^ cfu/ml) did not find differences in CH_4_ production, total GP, or total VFA concentration.

In this study, we observed that as *L. plantarum* product inclusion level increased, the greater were the negative effects on total VFA concentration (preliminary Exp. 1), and CO_2_ and CH_4_ production (preliminary Exp. 2), confirming that there is a limit to the *L. plantarum* inclusion level in order to avoid negative effects on ruminal fermentation. We also observed that greater *L. plantarum* concentrations used in our study (1.35 × 10^9^ cfu/g), as compared to other studies, was possibly around the upper limit of inclusion due to the negative effects on ruminal fermentation, as well as that these greater inclusion levels were possibly the reason for the greater effects observed in our study as compared to those reported by Ellis et al. (2016).

### Main Experiment

**Figure 1.**
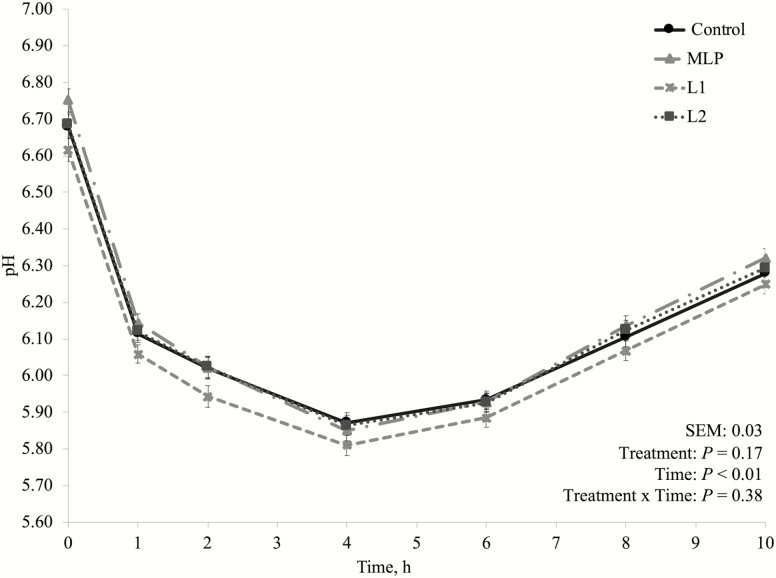
Dynamics of pH after the morning feeding in the main experiment (Exp. 3). Additive inclusion in the basal diet for each treatment: CTRL = control (no additive added); MLP = 0.01% of a mix of *L. acidophilus* and *P. freudenreichii*; L1 = 0.05% of *L. plantarum*; L2 = 0.10% of *L. plantarum*.

This experiment (Exp. 3) was designed to further evaluate the ruminal effects of *L. plantarum* as DFM in a high-producing dairy cows’ diet on true nutrient digestibility and N utilization. Treatments were selected based on the results of the preliminary Exp. 1 and 2: a control treatment without additive (CTRL), the treatment MLP as a positive control, and the treatments containing the two lowest inclusion levels of *L. plantarum* (L1 and L2). The two lowest inclusion levels of *L. plantarum* treatments were chosen based on the total VFA concentration and OM digestibility (Tables 3–5). Both of these variables were unaffected by the increasing in inclusion levels of *L. plantarum* treatments; therefore, the lowest amounts of the *L. plantarum* treatments were preferred. Unlike in the preliminary experiments, additives did not affect nutrient true digestibility (Table 6), and MLP did not differ in any variable compared to L1 and L2 throughout the experiment. As in the preliminary Exp. 1 that the final pH of the incubations was not affected by additive inclusion, in the main experiment, there was neither a treatment by time interaction for the pH of the first 10 h after the morning feeding (pool samples), nor a treatment effect (Fig. 1). Other studies evaluating the inclusion of pure lactic acid producing bacteria or in a mix with lactic acid utilizing bacteria have also reported that these DFM do not affect ruminal pH (Raeth-Knight et al., 2007; O’Brien et al., 2013).

**Table 6.** Effects of additives on nutrient true digestibility in a dual-flow continuous culture system (main experiment—Exp. 3)

	Treatments^1^					*P*-values^2^		
Item, %	CTRL	MLP	L1	L2	SEM	CTRL vs. additives	MLP vs. L1 and L2	L1 vs. L2
DM	55.6	55.0	54.4	56.0	1.83	0.72	0.88	0.30
OM	59.9	58.7	59.2	59.7	1.28	0.66	0.63	0.80
CP	53.6	51.9	52.3	51.5	1.72	0.35	0.99	0.70
NDF	68.3	64.7	67.0	66.5	1.97	0.33	0.39	0.85
Dietary starch	89.1	89.2	90.1	88.2	1.15	0.96	0.94	0.26

^1^Additive in the basal diet for each treatment (% of diet DM): **CTRL** = control (no additive added); **MLP** = 0.01% of a mix of *L. acidophilus* and *P. freudenreichii*; **L1** = 0.05% of *L. plantarum*; **L2** = 0.10% of *L. plantarum*.

^2^Significant differences were considered at *P ≤* 0.05, and a tendency was considered to be between *P >* 0.05 and *≤* 0.10.

Differently from preliminary Exp. 1 and 2 that the systems used evaluated short-term responses to ruminal fermentation (first 48 h), the main experiment was performed in a dual-flow continuous culture system, which allows 7 d for diet adaptation before 4 d of sample collections (Salfer et al., 2018). Also, despite the fact that diets used across all experiments were isocaloric and isonitrogenous, the basal diet in the main experiment contained a slightly greater proportion of fermentable nutrients (Table 1). Additives did not change total VFA concentration (Table 7) unlike preliminary Exp. 1. In our study, additives may have had a negative effect on ruminal fermentation in the first days (preliminary experiments), however, as the microbial community adapted to the additive those negative effects diminished.

**Table 7.** Effects of additives on VFA concentration in pool samples in a dual-flow continuous culture system (main experiment—Exp. 3)

	Treatments^1^					*P*-values^2^		
Item	CTRL	MLP	L1	L2	SEM	CTRL vs. additives	MLP vs. L1 and L2	L1 vs. L2
Total VFA, m*M*	119	115	116	113	4.29	0.33	0.93	0.60
VFA, % of total VFA								
Acetate	58.2	57.7	57.1	58.9	0.95	0.79	0.76	0.18
Propionate	16.4	16.6	17.2	16.0	0.46	0.68	0.97	0.06
Butyrate	15.6	14.8	14.8	15.0	0.60	0.34	0.86	0.80
Valerate	4.67	5.06	5.46	4.49	0.48	0.54	0.88	0.15
*Iso-*butyrate	1.29	1.33	1.23	1.32	0.05	0.99	0.39	0.16
*Iso-*valerate	3.55	3.64	3.80	3.86	0.20	0.36	0.44	0.84
Acetate:propionate	3.59	3.52	3.34	3.72	0.15	0.70	0.95	0.08
Total BCVFA^3^, m*M*	5.84	5.70	5.85	5.93	0.39	0.98	0.68	0.88
Lactate, m*M*	0.11	0.12	0.12	0.11	0.01	0.30	0.63	0.38

^1^Additive in the basal diet for each treatment (% of diet DM): **CTRL** = control (no additive added); **MLP** = 0.01% of a mix of *L. acidophilus* and *P. freudenreichii*; **L1** = 0.05% of *L. plantarum*; **L2** = 0.10% of *L. plantarum.*

^2^Significant differences were considered at *P ≤* 0.05, and a tendency was considered to be between *P >* 0.05 and *≤* 0.10.

^3^Total BCVFA = Branched-chain VFA (*Iso-*butyrate + *Iso-*valerate).

VFA = volatile fatty acids.

Similar to the preliminary Exp. 1 in which propionate concentration had a cubic response to *L. plantarum* treatments (L1 was greater than L2), in the main experiment, these treatments also tended to follow the same path and L1 tended to have a greater propionate molar proportion compared to L2 (*P =* 0.06). Due to the tendency for difference in propionate proportions, the A:P ratio also tended to be different between L1 and L2 treatments (*P =* 0.08) as the acetate concentration was not different across treatments. Although there may not be long-term negative effects from these products in terms of the ruminal environment, as explained in the preliminary Exp. 1 and two sections, *L. plantarum* may have antimicrobial effects against other bacteria (Zalán et al., 2005; O’Brien et al., 2013) and the greater inclusion may negatively affect propionate-producing bacteria.

**Figure 2.**
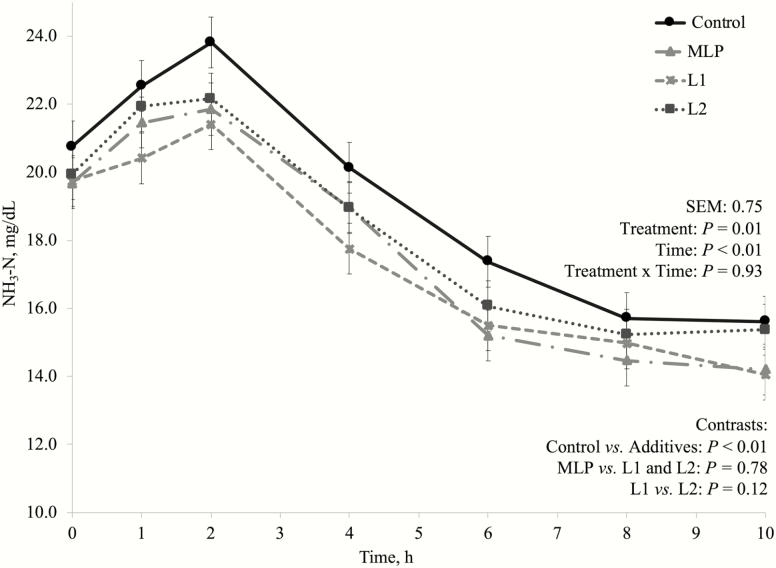
Dynamics of NH_3_-N concentration after the morning feeding in the main experiment (Exp. 3). Additive inclusion in the basal diet for each treatment: CTRL = control (no additive added); MLP = 0.01% of a mix of *L. acidophilus* and *P. freudenreichii*; L1 = 0.05% of *L. plantarum*; L2 = 0.10% of *L. plantarum*. Orthogonal contrasts: statistical differences were declared at *P* ≤ 0.05 or as a tendency if *P* > 0.05 and ≤ 0.10.

**Figure 3.**
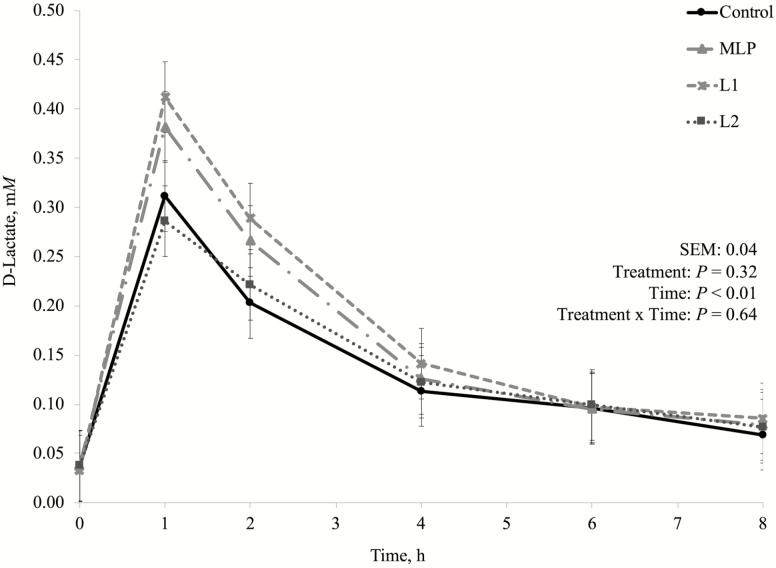
Dynamics of D-lactate concentration after the morning feeding in the main experiment (Exp. 3). Additive inclusion in the basal diet for each treatment: CTRL = control (no additive added); MLP = 0.01% of a mix of *L. acidophilus* and *P. freudenreichii*; L1 = 0.05% of *L. plantarum*; L2 = 0.10% of *L. plantarum*.

**Figure 4.**
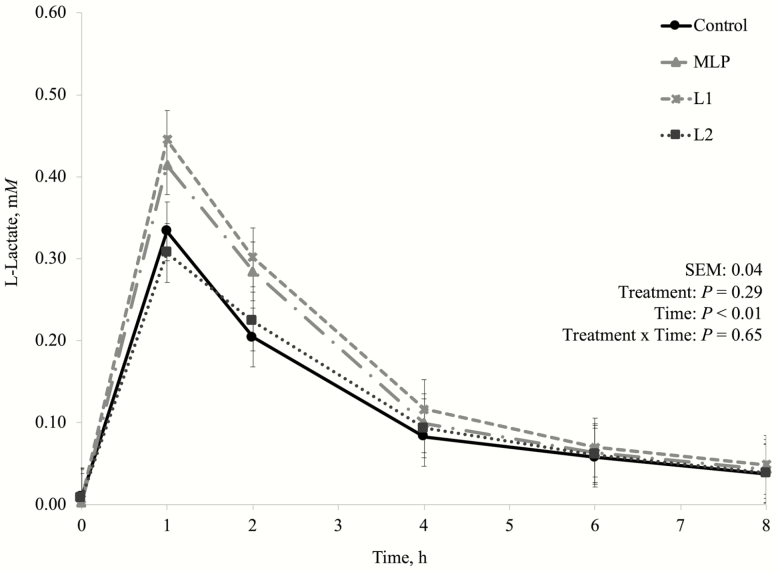
Dynamics of L-lactate concentration after the morning feeding in the main experiment (Exp. 3). Additive inclusion in the basal diet for each treatment: CTRL = control (no additive added); MLP = 0.01% of a mix of *L. acidophilus* and *P. freudenreichii*; L1 = 0.05% of *L. plantarum*; L2 = 0.10% of *L. plantarum*.

One of the concerns about using lactic acid producing bacteria as DFM for high-producing dairy cows is because these bacteria are commonly associated with their effects on silage fermentation. *Lactobacillus plantarum* as silage inoculants greatly reduce silage pH and preserves silage through lactate production (Muck et al., 2007; Contreras-Goveaa et al., 2013). This is one of the reasons why MLP product has been used by dairy producers instead, as it also has *P. freudenreichii* (lactic acid utilizing bacteria) in its composition. In this experiment, there was no treatment by time interaction nor a treatment effect on fermentation pH (Fig. 1), D-lactate (Fig. 2), L-lactate (Fig. 3), or total lactate concentrations (Fig. 4). As explained in the preliminary Exp. 1, diets that do not lead to high lactate production may not be negatively affected by lactic acid producing bacteria inclusion, since lactate is likely to be quickly metabolized by other bacterial groups (Weinberg et al., 2003), which may have happened in this experiment as well.

**Figure 5.**
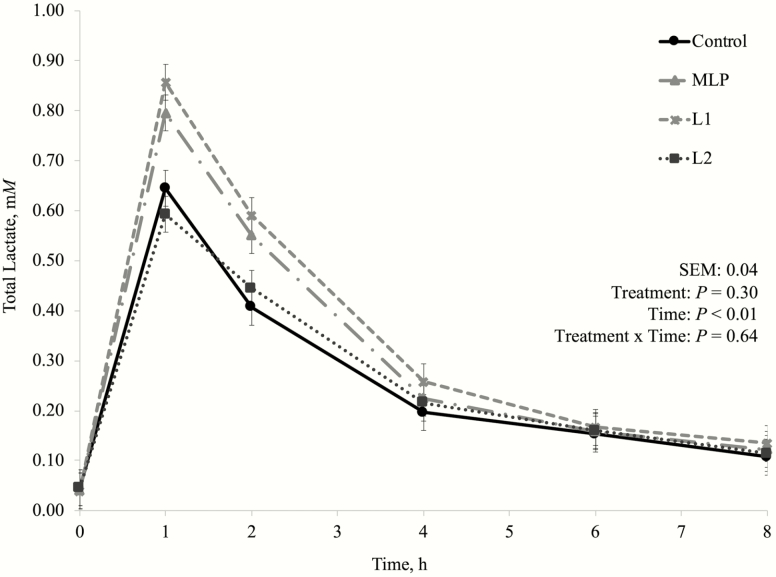
Dynamics of total lactate concentration after the morning feeding in the main experiment (Exp. 3). Additive inclusion in the basal diet for each treatment: CTRL = control (no additive added); MLP = 0.01% of a mix of *L. acidophilus* and *P. freudenreichii*; L1 = 0.05% of *L. plantarum*; L2 = 0.10% of *L. plantarum*.

All additives reduced NH_3_-N concentration in pool samples compared to the CTRL treatment (*P =* 0.05; Table 8) and tended to reduce total NH_3_-N flow (*P =* 0.07). For the dynamics of NH_3_-N concentration (mg/dL; Fig. 5), although there was no treatment by time interaction, there was a treatment effect (*P =* 0.01), and the additive treatments reduced NH_3_-N concentration (mg/dL) over time (*P <* 0.01). Studies evaluating MLP supplementation effects on ruminal fermentation in dairy cows are scarce and results are inconsistent. Raeth-Knight et al. (2007) reported no differences in NH_3_-N concentration, apparent total tract nutrient digestibility, and milk yield or composition when MLP was supplemented compared to a diet without supplementation. However, Boyd et al. (2011) reported increased apparent total tract CP digestibility, milk protein yield, and milk yield when dairy cows were supplemented with MLP. Contreras-Goveaa et al. (2013) reported that *L. plantarum* reduced NH_3_-N concentration and AA fermentation when used as a silage inoculant and increased microbial non-ammonia N and microbial biomass yield during ruminal fermentation. Together with our study, these other studies reinforce that similar DFM may affect the microbial community composition differently depending on where they are applied, and this might be the reason for the variable outcomes in different studies.

**Table 8.** Effects of additives on nitrogen utilization and bacterial glycogen in a dual-flow continuous culture system (main experiment—Exp. 3)

	Treatments^1^					*P*-values^2^		
Item	CTRL	MLP	L1	L2	SEM	CTRL vs. additives	MLP vs. L1 and L2	L1 vs. L2
NH_3_-N^3^, mg/dL	15.4	14.1	14.3	14.7	0.46	0.05	0.53	0.51
N flows, g/d								
Total N	2.73	2.76	2.79	2.75	0.06	0.59	0.92	0.62
NH_3_-N	0.63	0.59	0.58	0.60	0.02	0.07	0.74	0.38
NAN^4^	2.10	2.17	2.21	2.17	0.07	0.35	0.96	0.51
Bacterial-N	0.84	0.86	0.90	0.82	0.04	0.59	0.99	0.11
RDP-N^5^	1.75	1.71	1.71	1.69	0.05	0.37	0.91	0.72
RDP, % of N	58.0	56.6	56.8	56.1	1.55	0.36	0.95	0.71
RUP-N^6^	1.27	1.31	1.30	1.33	0.05	0.35	0.99	0.70
RUP, % of N	42.0	43.4	43.2	43.9	1.55	0.36	0.95	0.71
ENU^7^, %	47.9	50.3	53.2	48.6	3.00	0.42	0.87	0.25
Bacterial efficiency^8^	14.0	14.7	15.3	13.8	0.81	0.51	0.87	0.17
Bacterial glycogen, mg/d	104	127	122	111	15.0	0.32	0.53	0.56
Bacterial glycogen, % bacterial DM	1.07	1.19	1.18	1.18	0.15	0.54	0.92	0.99

^1^Additive in the basal diet for each treatment (% of diet DM): **CTRL** = control (no additive added); **MLP** = 0.01% of a mix of *L. acidophilus* and *P. freudenreichii*; **L1** = 0.05% of *L. plantarum*; **L2** = 0.10% of *L. plantarum.*

^2^Significant differences were considered at *P ≤* 0.05, and a tendency was considered to be between *P >* 0.05 and *≤* 0.10.

^3^NH_3_-N = ammonia nitrogen.

^4^NAN = non-ammonia nitrogen.

^5^RDP-N = rumen degraded protein nitrogen.

^6^RUP-N= rumen undegraded protein nitrogen.

^7^Efficiency of N use = g of bacterial N/g of available N.

^8^Bacterial efficiency = g of bacterial N/kg of OM truly digested.

Therefore, as we observed increased NH_3_-N utilization in the dual-flow continuous culture system and no differences in true nutrient digestibility (Table 6), bacterial-N, ENU, bacterial efficiency, and bacterial glycogen (Table 8), the products used in our study may have directly affected other microbial groups related to NH_3_-N use or AA fermentation and require further evaluation of these products and their effects on the microbial changes during ruminal fermentation.

## CONCLUSIONS

In conclusion, all additives tested had negative effects during short-term incubations in high-producing dairy cows’ diets (preliminary experiments), as well as no major differences were observed between yeast- and *Lactobacillus*-based additives. In the main experiment, which was designed to further evaluate *L. plantarum* effects on ruminal fermentation, additives tested (MLP, L1, and L2) did not have negative effects and reduced NH_3_-N concentration, indicating that these additives either reduce protein degradation or improve NH_3_-N utilization by ruminal microorganisms. In summary, although there are reservations regarding the use of LAB as DFM, none of our experiments detected changes in lactate concentration when these additives were used.
